# Development of a Multiplex PCR Method for Efficient Differential Diagnosis of Clinical Cases and Vaccine Immunization of Marek’s Disease

**DOI:** 10.3390/v18040471

**Published:** 2026-04-16

**Authors:** Wen-Kai Zhang, Man Teng, Lu-Ping Zheng, Bin Shi, Wei-Dong Wang, Gui-Xi Li, Yong-Xu Zhao, Zhen Yang, Zu-Hua Yu, Jun Luo

**Affiliations:** 1 Institute for Animal Health & UK-China Centre of Excellence for Research on Avian Diseases, Henan Academy of Agricultural Sciences, Zhengzhou 450002, China; 2 Laboratory of Functional Microbiology and Animal Health, College of Animal Science and Technology, Henan University of Science and Technology, Luoyang 471003, China; 3 Shangqiu Center for Animal Quarantine and Disease Control and Prevention, Shangqiu 476100, China; 4 Scientific Support and Investigation Unit, Ceva Animal Health, Hangzhou 310018, China; 5 Jinyu Youbang Biotechnology (Jiangsu) Co., Ltd., Yangzhou 225007, China

**Keywords:** Marek’s disease virus, multiplex PCR, viral infection, vaccination, differential diagnosis

## Abstract

Marek’s disease (MD), caused by pathogenic Marek’s disease virus serotype 1 (MDV-1), is one of the most important avian immunosuppressive and neoplastic diseases and has led to huge economic losses to the poultry industry worldwide. Rapid and accurate clinical diagnosis is of great significance for efficient control of the disease. Herein, we have established a multiplex PCR (mPCR) method to simply differentiate all of the three types of MDV, using five specific primers targeting to MDV-1 oncogene meq or MDV-2 and MDV-3/HVT *gB* genes. Simultaneously, it can detect any type of virulent or vaccine MDV strains in one PCR reaction, with amplicons of the short (S) and long (L)-meq of MDV-1 strains, and the *gB* of MDV-2 and HVT vaccine strains. Non-specific amplifications of avian leukosis virus (ALV), reticuloendotheliosis virus (REV), or fowl adenovirus virus 4 (FAdV-4) were not observed, indicating a good specificity of this method. A total of 522 clinical samples of tumor-bearing or suspected diseased birds collected from 30 poultry farms were detected. The results demonstrated that the newly developed mPCR method accurately detected and differentiated epidemic MDV-1 infections and vaccine strains, and provided nearly 100% consistency for detecting clinical wild-type infections compared with conventional PCR amplification of the meq gene. Collectively, our data has provided a highly efficient method for early differential diagnosis of MD clinical cases, virus identification and future evaluation of vaccination efficacy in healthy chicken flocks, which would be meaningful for efficient control of the disease.

## 1. Introduction

Marek’s disease (MD) is an important disease of poultry caused by oncogenic Marek’s disease virus (MDV) and characterized by serious immunosuppression and T-cell lymphomas [1]. It has caused huge economic losses to the poultry industry worldwide, with an estimated annual direct economic loss exceeding $1 billion [2]. Historically, the causative agent MDV had been classified into three serotypes, including serotype 1 (MDV-1), serotype 2 (MDV-2) and serotype 3 (MDV-3), which is also called herpesvirus of turkeys (HVT). In the latest virus taxonomy profile, these viruses have been sequentially reclassified as *Gallid alphaherpesvirus* 2 (GaAHV-2), *Gallid alphaherpesvirus* 3 (GaAHV-3) and *Meleagrid alphaherpesvirus* 1 (MeAHV-1), respectively [3]. Only the MDV-1 isolates are pathogenic and oncogenic to chicken hosts, while MDV-2 and HVT viruses are non-pathogenic and usually used as vaccine strains [4]. Vaccination is the most effective strategy for the control of disease, and the most widely used commercial MD vaccine strains are usually natural non-pathogenic isolates or artificially attenuated strains, such as MDV-1 strains CVI988/Rispens [5,6,7], 814 [8], MDV-2 strain SB-1 [9], and HVT strain FC-126 [10,11]. In the later part of the 20th century, the monovalent and bivalent vaccines such as HVT+SB-1, CVI988+HVT and 814+HVT have made a significant contribution to the efficient prevention and control of MD.

In the past five decades, under the long-term and highly immune pressure of MD vaccination, the virulence of epidemic MDV strains has persistently evolved and significantly increased [12,13]. Notably, the newly emerged very virulent plus MDV (vv+MDV) and particularly the hypervirulent variants of MDV (HV-MDV) have been demonstrated to be capable of significantly evading the immune protection conferred by currently used commercial MD vaccines [14,15]. Most recently, frequent outbreaks of MD cases have occurred throughout the world, especially in Asian countries [16,17,18,19]. It is well known that once infected, both the virulent and vaccine strains of MDV can establish a lifelong latent infection in chicken hosts, resulting in a huge difficulty in clinical diagnosis to differentiate the vaccination and field infection of viruses in poultry farms. Thus, a rapid, easy and accurate method for the early differential diagnosis of MD cases and vaccine immunization is urgently needed to be developed for efficient control of the disease.

The classical methods for clinical diagnosis of MD mainly include virus isolation, agar gel precipitation (AGP) and histopathological diagnosis. In recent years, molecular biological techniques have been well developed and applied in the diagnosis of infectious diseases. Compared to traditional immunological methods, the diagnostic techniques based on nucleic acids amplification have displayed excellent features of high throughput, higher sensitivity and higher specificity. For example, even low-abundance of viral particles or target genes in clinical samples can be easily and precisely detected by polymerase chain reaction (PCR) and real-time quantitative PCR (qPCR), which have been employed as highly accurate and sensitive methods to detect both virulent MDV and/or vaccine strains [20,21,22,23,24,25]. However, in the same PCR reaction, none of the previously developed methods can simultaneously detect and distinguish all three serotypes of MDV. Herein, we have developed a multiplex PCR (mPCR) method for differential detection of any virulent MDV strains and/or classical vaccine strains of all three serotypes, and have successfully applied it in the detection of a large number of clinical samples collected from tumor-bearing chicken flocks and poultry farms. Our data provide a novel tool for the early differential diagnosis of clinical MD cases, virus isolation and identification, and future evaluation of the vaccination efficacy of MD vaccines.

## 2. Materials and Methods

### 2.1. Ethics Statement

The clinical sample collection and experimental protocol were reviewed and approved by the Laboratory Animal Management Committee of Institute of Animal Health (IAH), Henan Academy of Agricultural Sciences (HAAS, Zhengzhou, China), following the protocols of the Laboratory Animal Guidelines for the Ethical Review of Animal Welfare permitted by State Administration for the Market Regulation and Standardization Administration of China (permit no. GB/T 35892-2018).

### 2.2. Viruses and Cells

The very virulent (vv) MDV strain GX0101 was gifted by Prof. Zhi-Zhong Cui (Shandong Agricultural University, Tai’an, China). The CVI988 and HVT vaccines were purchased from Boehringer Ingelheim (Shanghai, China). The SB-1, avian reticuloendotheliosis virus (REV) strain HNGS206 [18], avian leukosis virus (ALV) strain HNXZ2 [19], and fowl adenovirus serotype 4 (FAdV-4) strain ZZ were maintained in our lab (IAH, HAAS, Shanghai, China). Primary chicken embryo fibroblast (CEF) monolayers were prepared from 9-day-old specific pathogen-free (SPF) embryos (Beijing Boehringer Ingelheim Vital Biotechnology, Beijing, China). MDV was passaged in CEFs maintained in M199 medium (Thermo Fisher Scientific, Waltham, MA, USA) containing 1% fetal bovine serum (Gibco, Waltham, MA, USA) plus penicillin-streptomycin (Ncmbio, Suzhou, China) and incubated at 38.5 °C in a 5% CO_2_ incubator.

### 2.3. Primer Design and Synthesis

The conserved sequences of meq or *gB* genes from 18 reference MDV viruses, including 16 MDV-1 strains, MDV-2 strain SB-1 and MDV-3/HVT strain FC-126, listed in Table S1, were analyzed using the DNAMAN software v6.0 (Lynnon Biosoft, San Ramon, CA, USA). The specific oligos, including 18 primers for MDV-1, 8 primers for SB-1 and 13 primers for HVT targeting the 5′ or 3′ ends of target meq or *gB* genes (Table S2), were designed using Primer Premier 5.0 (Premier Biosoft International, Palo Alto, CA, USA). All the oligos were synthesized by Sangon Biotech (Shanghai, China).

### 2.4. Primer Test and Positive Control Plasmids

The specificity of all designed primers listed in Table S2 was first tested by PCR amplification. Briefly, the genomic DNAs of MDV strains GX0101, CVI988, SB-1, and HVT were separately extracted using the DNA extraction kit (TIANGEN, Beijing, China), and used as templates for amplifications of MDV-1 short/long meq (S-meq/L-meq), SB-1 *gB*, or HVT *gB* genes. The 20 μL PCR reaction mixtures contain 10 µL of 2× Easy Taq PCR SuperMix (+dye) (TransGen Biotech, Beijing, China), 1.0 μL DNA (100 ng/μL), 0.5 μL of primer sets (10 μM each), and 8.0 µL of ddH_2_O. The procedure was set as 94 °C for 4 min, 30 cycles of 94 °C for 30 s, 60 °C for 30 s, and 72 °C for 1 min; and a final extension at 72 °C for 5 min and finally maintained at 4 °C. PCR products were analyzed by 1% agarose gel electrophoresis to judge the primer specificities. Furthermore, using confirmed specific primers, the meq and *gB* genes from MDV strains GX0101, CVI988, SB-1 or HVT were separately amplified and cloned into the pMD19-T vector (TaKaRa, Shiga, Japan) to serve as positive control plasmids for the next experiments.

### 2.5. Establishment of a Multiplex PCR Method

All the available primers specific to MDV-1 meq, SB-1 or HVT *gB* genes were randomly combined, and each PCR amplification was performed in one reaction. PCR procedures were set up as described above, and products were electrophoresed to evaluate the specificity and sensitivity of different primer combinations. Then, the optimal combinations were chosen to establish the mPCR method. For further experiments, the positive control plasmids pMD19-T-L-meq, pMD19-T-S-meq, pMD19-T-SB-1gB and pMD19-T-HVTgB (10 ng/μL each) were mixed at a ratio of 10:10:1:1, and each of the 1 μL mixture was used as the DNA template for multiplex PCR amplification. As listed in Table 1, the meq-specific primers meq-2F/2R set at 5 μM, combining with gB-specific primers SB-1-1F/SB-1-1R/HVT-8R set at 1, 2, 3, 4 or 5 μM each, were separately used to determine the optimal concentration of primer combinations. The procedure was adjusted based on PCR, of which the annealing temperature gradient (55 °C, 57.5 °C, 60 °C, 62.5 °C, 65 °C) was set based on the GC contents and Tm values. Finally, all the products of mPCR amplifications were analyzed by 1% agarose gel electrophoresis.

### 2.6. Determination of the Specificity and Sensitivity of mPCR

The specificity of mPCR was separately determined using the viral DNA or cDNA of MDV, ALV, REV and FAdV-4 viruses as templates (10 ng/μL). Simultaneously, 1.0 μL viral DNA of GX0101, CVI988, SB-1, HVT or a mixture of four viral DNAs (10 ng/μL each) was used as templates for the mPCR amplification. The reaction program was set as follows: 94 °C for 4 min; 30 cycles of 94 °C for 30 s; 60 °C for 30 s; and 72 °C for 1 min; extension at 72 °C for 5 min, and finally maintained at 4 °C. The products were analyzed by 1% agarose gel electrophoresis to assess the specificity of mPCR. The positive control plasmids pMD19-T-L-meq, pMD19-T-S-meq, pMD19-T-SB-1gB, and pMD19-T-HVTgB were each diluted to a concentration of 10 ng/μL to serve as DNA templates. The copy number (copies/μL) was calculated using the formula: copy number (copies/μL) = NA (copies/mol) × concentration (g/μL)/MW (g/mol), where NA is Avogadro’s number, and MW is the base number times 340. Each plasmid was diluted 10-fold with EB (elution buffer) and used as a template for mPCR amplification. Sensitivity of mPCR was finally assessed using 1% agarose gel electrophoresis.

### 2.7. Application of mPCR for Diagnosis of Clinical MD Cases

A total of 522 clinical samples, including 251 livers and 271 spleens as listed in Table 2, were collected from 30 tumor-bearing chicken flocks during 2020–2022 as previously described [19]. The species of birds sampled mainly included Hyline Brown, Jinghong and some local chickens such as Liangfenghua, Partridge chicken and Muyuan Red, with onset ages of disease ranging from 17 to 200 days. The total DNA of spleen and liver samples was separately extracted, and each was diluted to a concentration of 100 ng/µL for further detection of virus infection by mPCR amplification as described above. To evaluate the accuracy of mPCR for the diagnosis of MD cases, as shown in Table 3, a comparison experiment was performed by PCR amplification of MDV-1 meq, using the same approach and same batches of 100 splenic samples randomly selected from tumor-bearing chicken flocks as previously reported [19]. Briefly, the PCR reaction was 20 µL, including 10 µL of 2×Easy Taq PCR SuperMix (+dye) (TransGen Biotech, China), 0.5 µL of primers MDV-meq-F/R (10 µM each), 1.0 µL of DNA template (100 ng/µL), and 8.5 µL of ddH_2_O. The reaction procedure for amplification of the meq gene was set as 94 °C for 4 min, 30 cycles of 94 °C for 1 min, 60 °C for 30 s and 72 °C for 1 min, extension at 72 °C for 10 min, and finally maintained at 4 °C. The amplicons were finally analyzed by 1% agarose gel electrophoresis.

## 3. Results

### 3.1. Specificity of PCR Primers Designed for Amplifying the Viral *meq* and gB Genes

**Figure 1:**
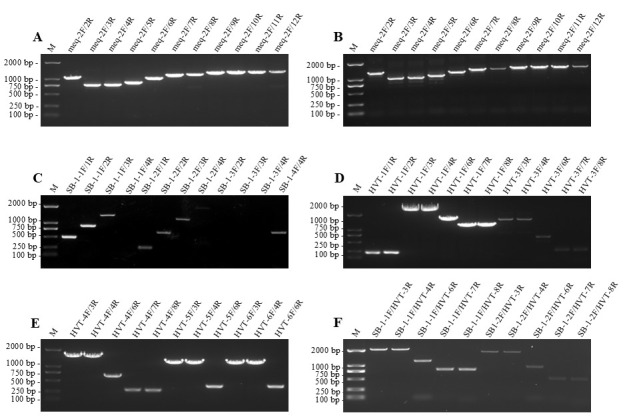
PCR amplification of meq and *gB* genes for testing the specificities of different primer combinations. (**A**) GX0101 virus was used as a template for PCR amplification of *meq* genes; (**B**) CVI988 virus was used as a template for PCR amplification of meq genes. (**C**) The SB-1 virus was used as a template for PCR amplification of *gB* genes; (**D**–**F**) the HVT virus was used as a template for PCR amplification of *gB* genes. The primer pair is abbreviated, such as meq-2F/2R, shortened from meq-2F and meq-2R. M, DNA marker.

The oncogene meq was preferentially selected as a key target for detecting and differing the virulent and attenuated strains of MDV-1. As listed in Table S2, a total of 18 upstream (5′) or downstream (3′) primers were designed and formed 77 PCR combinations to target the meq genes. Using each of them, the conventional PCR amplification was performed with the vvMDV strain GX0101 and the vaccine strain CVI988 to preliminarily test the primer performance and specificity. For both MDV-1 strains, as demonstrated in Figure 1A,B and Figure S1, the upper primers meq-2F, meq-5F, meq-7F, meq-8F and meq-9F were significantly more specific than the other primers, such as meq-3F and meq-6F that are matched by the corresponding bottom primers.

To detect the *gB* genes of MDV-2 and HVT, a total of 23 oligos forming 45 PCR combinations, as listed in Table S2, were each preliminarily tested. The results showed that for the MDV-2 strain SB-1, the PCR combinations containing upstream primer SB-1-1F produced the more abundant products and yielded the expected size. In contrast, the other PCR combinations containing primers SB-1-2F, -3F and -4F generated a lower abundance of products or failed to amplify specific bands (Figure 1C). For the HVT virus, amplicons with the expected size were specifically amplified by most of the combined PCR primer pairs (Figure 1D,E). Notably, primer combinations using SB-1-1F and -2F as upper primers and a part of HVT gB-specific oligos (such as HVT-3R, -4R, -6R, -7R and -8R) as bottom primers also worked well and the specific bands were perfectly amplified from HVT strain FC-126, of which the upper primer SB-1-1F was significantly better than SB-1-2F (Figure 1F).

Based on these data, the specific primer combinations, including meq-2F/2R, SB-1-1F/1R, and SB-1-1F/HVT-8R, listed in Table 1 and targeting the outermost regions of relevant target genes, were separately chosen for amplifying the MDV-1 meq, SB-1 or HVT *gB* genes to construct positive control plasmids. The constructed plasmids pMD19-T-L-meq, pMD19-T-S-meq, pMD19-T-SB-1gB and pMD19-T-HVTgB were further identified by PCR amplifications with primer pairs meq-2F/2R, SB-1-1F/1R and SB-1-1F/HVT-8R, and the electrophoresis analysis showed that all four plasmids produced specific bands in a clear view and correct sizes, without non-specific PCR products (Figure S2).

### 3.2. Optimal Primer Combination and Reaction Conditions for mPCR Amplification

**Figure 2:**
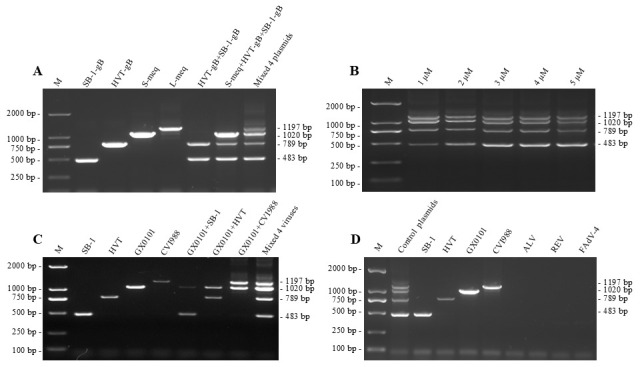
Specificity of primer combination meq-2F/2R+SB-1-1F/1R+HVTgB-8R determined by mPCR amplification. (**A**) Single, double or multiplexed positive control plasmids were used as DNA templates; (**B**) optimization of concentrations of primers SB-1-1F, SB-1-1R and HVTgB-8R used for mPCR amplification; (**C**) single, double or multiplexed MDV viruses were used as DNA templates; (**D**) non-specificity to the other viruses determined by mPCR amplification. M, DNA marker; ALV, avian leucosis virus; REV, reticuloendotheliosis virus; FAdV-4, fowl adenovirus virus 4.

According to the primary PCR amplification tests, primer pairs with high specificity and high product yield to generate amplicons with sizes ranging from 200 to 1500 bp were preferentially selected for the development of mPCR methods. For example, the primer pairs meq-2F/2R, meq-5F/7R or meq-7F/7R, together with SB-1-1F/1R and HVT-8R, were cross-combined to form three sets of primer mixtures for developing mPCR methods, and the unique or mixed positive control plasmids were used as DNA templates for testing. As demonstrated in Figure 2A, the preliminary PCR results showed that primer combination meq-2F/2R+SB-1-1F/1R+HVT-8R displayed the highest specificity and uniformly distributed bands of expected sizes of all target genes. However, the other two primer combinations, meq-5F/7R+SB-1-1F/1R+HVT-8R and meq-7F/7R+SB-1-1F/1R+HVT-8R, were both observed to be not effective for mPCR amplification and displayed non-specific products (Figure S3A,B). The data indicated that the mixed primers meq-2F/2R+SB-1-1F/1R+HVT-8R, as listed in Table 1 and schematized in Figures S4–S6, are possibly an optimal combination for specific detection and differentiation of all three serotypes of MDVs in the same PCR reaction system.

The optimal primer ratio used for mPCR was further determined using the mixed positive control plasmids as templates, including pMD19-T-L-meq, pMD19-T-S-meq, pMD19-T-SB-1gB, and pMD19-T-HVTgB. It has been shown that when the concentration of meq-2F/2R primers was set at 10 μM (0.5 μL each), four specific bands were all amplified as expected across a range of ratios of the other three primers. As demonstrated in Figure 2B, the amplicons appeared clearest and mostly uniform when each of the concentrations of SB-1-1F, SB-1-1R and HVT-8R ranged from 3 to 5 μM (final concentration). Thus, the final optimized mPCR reaction system consisted of 2× Easy Taq PCR SuperMix (+dye) 10 μL, meq-2F/2R (10 μM each) 0.5 μL each, SB-1-1F/1R (10 μM) 0.3 μL each, HVT-8R (10 μM) 0.3 μL, and ddH_2_O added to 20 μL. Furthermore, a gradient annealing temperature from 55 °C to 65 °C was performed, but interestingly, all of them worked well and produced specific bands as expected, without a significant difference (Figure S3C).

Collectively, the optimal system and reaction conditions for mPCR amplification were finally set as follows: 2× Easy Taq PCR SuperMix (+dye) 10 μL, primers meq-2F/2R (10 μM each) 0.5 μL each, SB-1-1F/1R and HVT-8R primers (10 μM each) 0.3 μL each, plus ddH2O to 20 μL. The plasmids pMD19-T-L-meq, pMD19-T-S-meq, pMD19-T-SB-1gB and pMD19-T-HVTgB (10 ng/μL each) mixed at a ratio of 10:10:1:1 (*v*:*v*:*v*:*v*) serve as positive controls. The procedure was set as: 94 °C for 4 min, 30 cycles of 94 °C for 30 s, 60 °C for 30 s, and 72 °C for 1 min; and a final extension at 72 °C for 5 min and maintained at 4 °C. The PCR products were finally analyzed by 1% agarose gel electrophoresis.

### 3.3. Specificity and Sensitivity of Newly Developed mPCR Method for Detecting MDV

The specificity of the mPCR method was first evaluated using viral DNA collected from CEF cultures infected with different serotypes of MDV reference strains, including virulent MDV-1 strains GX0101 and commercial vaccine strains CVI988, SB-1 and HVT FC-126. As demonstrated in Figure 2C, mPCR amplifications produced all the specific bands in expected sizes, regardless of whether the DNA template was a single, double or triple-mixture of distinct serotypes of MDVs. Furthermore, the viral DNA of MDV, ALV, REV, and FAdV-4 was used as templates for testing the non-specificity of mPCR amplification. The results showed that only the expected MDV-specific bands were amplified from the samples of SB-1, HVT, GX0101 or CVI988-infected CEFs, while no bands were amplified in CEFs infected with ALV, REV or FAdV-4 viruses (Figure 2D).

**Figure 3:**
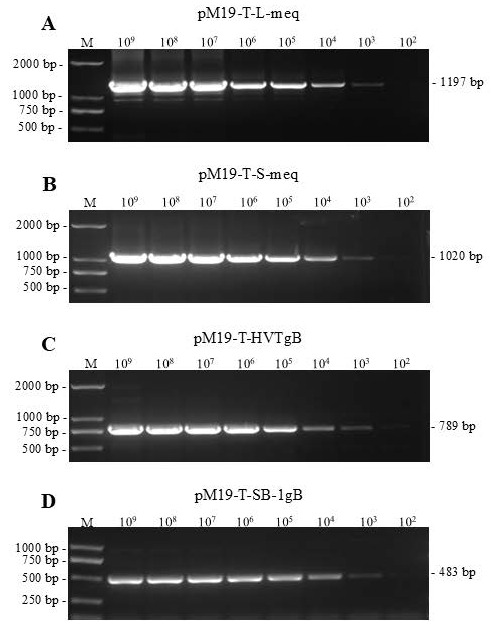
Sensitivity of mPCR to detect positive control plasmids containing target genes from different serotypes of MDV. (**A**) Plasmid pMD19-T-L-Meq; (**B**) plasmid pMD19-T-S-meq; (**C**) plasmid pMD19-T-HVTgB; (**D**) plasmid pMD19-T-SB-1gB. M, DNA marker.

The positive control plasmids pMD19-T-L-meq, pMD19-T-S-meq, pMD19-T-SB-1gB and pMD19-T-HVTgB were separately and serially diluted in 10-fold to generate a standard series of DNA templates ranging from 10^9^ to 10^2^ copies/μL and used for detecting the sensitivity of mPCR. Interestingly, it has been demonstrated that for all four templates, the detectable limitations were all at a limit concentration of 10^3^ copies/μL (Figure 3). Taken together, it indicates that the newly developed mPCR method can specifically detect, identify and differentiate all three serotypes of MDVs, either virulent strains or attenuated/avirulent vaccine strains, with high specificity and sufficient sensitivity.

### 3.4. Clinical Application of mPCR for Diagnosis of MD Cases Differentiating Vaccination

**Figure 4:**
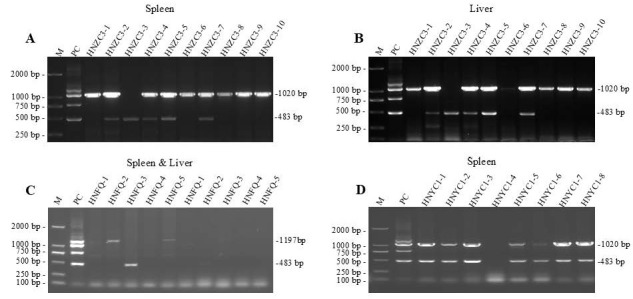
MDV infection in clinical samples collected from different poultry farms, detected by mPCR. (**A**,**B**) Splenic and liver samples collected from a suspected MD case occurred in the 3rd poultry farm in Zhecheng, Henan (HNZC), with sample nos. 1~10; (**C**) suspected MD case occurred in Fengqiu, Henan (HNFQ), with splenic or liver sample nos. 1~5; (**D**) suspected MD case occurred in the 1st poultry farm, Yucheng, Henan (HNYC), with splenic sample nos. 1~8. M, DNA marker; PC, positive control plasmids.

The newly developed mPCR method was further applied to detect virus infections in suspected MD cases, including 522 clinical samples (251 livers and 271 spleens) collected from the tumor-bearing chicken flocks in 30 poultry farms (Table 2). As expected, infection of epidemic MDV-1 isolates was positively detected in most of the clinical cases, together with detectable MD vaccine strains in a part of the samples, of which the representative results of mPCR amplifications are demonstrated in Figure 4, whereas the other electrophoresis data are mainly displayed in Figure S7.

In details, as shown in Table 2, the statistical data revealed that in individual birds the positive infection rate of wild type MDV-1 viruses (represented by S-meq) in spleens and livers were 62.4% (169/271) and 58.6% (147/251) respectively, indicating similar infection rates between two tissues, accumulated in a total positive rate of 60.5% (316/522) and contributing to an accumulative flock positive rate of 86.7% (26/30). Interestingly, the MDV-2 vaccine strain SB-1 was also predominantly detectable in 55.9% (292/522) individual birds, with a flock positive rate of 90% (27/30). For the other two vaccine strains, CVI988 and HVT FC-126, the positive rates were both significantly lower, with only displayed individual positive rates of 2.5% (13/522) and 0.2% (1/522), accompanied by flock positive rates of 13.3% (4/30) and 3.3% (1/30), respectively.

To further evaluate the accuracy of mPCR for the diagnosis of clinical MD cases, a comparison between mPCR and conventional PCR was performed, and the same batch of 100 splenic samples randomly selected from 10 tumor-bearing chicken flocks was used for detecting MDV-1 meq genes. As shown in Table 3, the cumulative positive rates of S-meq, L-meq and S-meq+L-meq of MDV-1 detected by mPCR were 75% (75/100), 5% (5/100), and 80% (80/100), while the corresponding data detected by conventional PCR were 76% (76/100), 5% (5/100), and 81% (81/100), respectively. It indicates that mPCR displays a high consistency with traditional PCR and is valuable for the diagnosis of clinical MD cases.

## 4. Discussion

It is well known that the occurrence of tumors can be effectively prevented or significantly decreased by the MD vaccine, but it cannot block infection and transmission of epidemic MDV strains, which makes the early differential diagnosis of MD cases challenging in clinical practice. The rapid detection of co-infections of causative agents of avian immunosuppressive and neoplastic diseases, efficient differentiation of failed virus infections and vaccine immunizations, and the epidemiological surveillance of MDV are all dependent on the development of a next-generation of molecular biological techniques. Under laboratory conditions, different serotypes of MDV involved in clinical cases can be characterized by virus isolation, PCR amplification, DNA sequencing, and even immunoassays such as immunofluorescence assay (IFA) using serotype-specific antibodies [26,27]. If needed, qPCR analysis can be employed for the quantitative monitoring of MDV. Previously, based on the Mismatch Amplification Mutation Assay-PCR (MAMA-PCR), a method was developed utilizing two sets of specific primers designed to target a single nucleotide polymorphism (SNP) associated with the H19 epitope within the MDV-1-specific *pp38* gene, enabling the quantitative differentiation of the CVI988 vaccine strain from other MDV-1 strains [21]. Similarly, a qPCR method for distinguishing CVI988 and virulent MDV-1 strains based on sequence differences in the meq genes was also established [23]. For field-based diagnosis, loop-mediated isothermal amplification (LAMP) and portable recombinase polymerase amplification (RPA) have been developed for use in point-of-care veterinary settings [28,29,30]. However, currently used methods for detecting MDV still exhibit notable limitations, including the relatively high costs of reagents and expensive instruments that limit the accessibility of techniques such as qPCR and RPA. Meanwhile, although cost-effective, LAMP is susceptible to false-positive results. Thus, an ideal platform for the detection and differentiation of distinct serotypes of virulent and/or vaccine MDV strains, balancing accuracy, cost-effectiveness, multiplexing capability, and practicality for clinical diagnosis, is urgently needed.

Relatively, the mPCR method is a more efficient, simple and low-cost multiplex nucleic acid detection technique derived from traditional PCR, which can be applied for the simultaneous detection and differentiation of multiple avian pathogens [31]. Compared to LAMP technology, high-throughput detection can be achieved by mPCR, and the probability of false positives caused by cross-contamination is significantly lower. Compared to technologies such as qPCR, RPA and even oligonucleotide microarray [32], detection of multiple targets can be simultaneously realized in one tube by mPCR, which can be easily operated by ordinary laboratory personnel without expensive equipment. Relatively, mPCR also has some shortcomings, such as longer amplification time than RPA and lower sensitivity than qPCR and oligonucleotide microarray. However, it is clear that greater advantages can be offered by mPCR in veterinary clinical diagnosis, characterized by the low cost, high specificity, sufficient sensitivity, and especially competent in distinguishing field MDV infection and vaccine immunization.

To address the urgent need for early diagnosis of MD cases, a conventional (end-point) mPCR method, capable of simultaneous serotype discrimination and vaccine/field differentiation, and optimized for cost-effectiveness and routine diagnostic laboratories, has been established based on the optimization of 39 primers that formed 122 combinations targeting MDV-1 meq or SB-1/HVT *gB* genes. It has been shown that the primer design and screening played a critical role in the success of developing the mPCR method, which displayed a high specificity to MDV rather than the other commonly co-infected pathogens such as ALV, REV and FAdV-4. The limit of detection (LOD) of the newly developed mPCR method was determined as low as 10^3^ copies of viral DNA. Recently, a TaqMan-probe-based quadruplex real-time PCR was developed for the discrimination and quantification of MDV vaccine strains and field strains, with a reported LOD of 10 copies of plasmid DNA [33]. However, in the same work, LODs of comparative PCR assays for conventional detections of CVI988 and virulent MDV were both 10^4^ copies, whereas for HVT, it was 10^6^ copies. It indicates that although it is lower than qPCR, the sensitivity of mPCR was significantly higher than the previous report, which may be sensitive enough for clinical diagnosis. However, using meq amplicon length as a sole discriminator is a proxy marker rather than an absolute indicator of virulence. A recent high-fidelity sequencing study has demonstrated that the extensive meq isoforms (VVL-Meq, VL-Meq, L-Meq, Meq, S-Meq and VS-Meq) and some virulent MDV strains (e.g., HPRS-B14) also share the L-Meq pattern [34]. Furthermore, virulent strains may harbor meq deletions or length polymorphisms, and recombinants can complicate interpretation, implying that further confirmation by gene cloning, DNA sequencing and even animal experiments may be needed in ambiguous cases.

Practically, the mPCR method presently established has been well characterized by substantially lower costs and greater suitability for routine clinical MD diagnosis. Our results showed that the flock positive rate of epidemic MDV-1 infection in 30 tumor-bearing poultry farms detected by mPCR was 86.7% (26/30), accompanied by the positive rates of 62.4% (169/271) in spleens and 58.6% (147/251) in livers that were very similar to previously reported data [19]. A comparative experiment performed on the same batch of 100 clinical splenic samples also showed a nearly 100% consistency between mPCR and traditional PCR, further suggesting the newly developed mPCR method as a suitable approach for the detection of MDV in clinically suspected cases. Interestingly, a highly positive co-infection of the SB-1 vaccine strain in most MD cases was also detected by mPCR, and a part of these isolates has been confirmed by DNA sequencing. Although it is difficult to explain the underlying reasons, such as the persistence of vaccine virus, environmental contamination, and/or detection of residual vaccine DNA, it has been a common phenomenon happening in field MDV prevalent poultry farms, not only in the present investigated central China but also in the southeast province of Fujian (Yanping Zhang, personal communication). Whether the prevalent SB-1 reduces the immunity of commercial MD vaccines or the immune failure promotes the prevalence of SB-1 needs to be further studied.

Except for SB-1, the mPCR presently developed has also successfully detected positive infection of vaccine strains CVI988 and/or HVT in a part of clinical MD cases, implying that in the same tube, it can effectively differentiate distinct serotypes of MDV. Recently, outbreaks of MD have frequently happened in vaccinated chicken flocks worldwide, especially in Asian countries [17]. With significantly increased virulence, the emergence of vv+MDV and particularly the HV-MDV has been confirmed to significantly overcome the protection of most widely used MD vaccines, including monovalent CVI988, 814, HVT and bivalent CVI988+HVT vaccines [14,15]. Currently, inoculation of two types and/or double-doses of the MD vaccines, such as CVI988 and CVI988+HVT, has been performed in many breeder farms in China and achieved a good immune protection against epidemic MDV strains (personal communication). However, for most commercial laying hens and broilers raised for a long cycle, limited by cost, only a single dose of the MD vaccine is commonly used, but could not provide effective protection, usually resulting in immune failures in some farms and causing serious losses [19]. Under this situation, evaluating the immune efficacy of MD vaccines that are produced with any serotype of vaccine strains has become an important strategy for the efficient prevention and control of MD. Since MDV mainly induces T-cell immune responses and lacks suitable humoral immunity indicators, assessing the in vivo proliferation of virus in birds post vaccination can effectively evaluate the positive rate, uniformity and effectiveness of MD vaccines in chicken flocks. Undoubtedly, the mPCR method established in this study provides an ideal technical approach for this work.

In conclusion, we have developed a conventional but meaningful mPCR method for early differential diagnosis of MD clinical cases, virus identification and future evaluation of vaccination efficacy in healthy chicken flocks, which would be contributory for efficient control of the disease. In the future, for different host species and MD vaccines, the optimal type of samples, collection time points and correlation between vaccination status and final protection efficacy still need further research. Validation of the sensitivity and specificity changes based on age, days post-vaccination/challenge, presence of tumors or in latency during various stages will also demonstrate the reliability of the mPCR method under different conditions. Furthermore, in our experience, a higher sensitive, multiplex real-time fluorescent quantitative differentiation and diagnostic technique based on qPCR deserves to be further studied.

## Figures and Tables

**Table 1:** Primers used for the multiplex PCR amplification of different types of MDV.

Serotype	Target	Primer	Type	Sequence (5′-3′)	Length (nt)	Genomic Location	Amplicons (bp)
MDV-1	S-meq/L-meq	meq-2F	5′	ATGTCTCAGGAGCCAGAGCCGGGCGCTAT	29	133603–133631 ^a,b^	1020/1197
		meq-2R	5′	TCAGGGTCTCCCGTCACCTGGAAACCACCA	30	134593–134622 ^a,b^	
MDV-2	SB-1 gB	SB-1-1F	5′	TGCCCCATACCGTTGAACAATTCCGCC	27	59051–59077 ^c^	789
		SB-1-1R	5′	GCAGAATTCCATGAGGGTCGC	21	59513–59533 ^c^	
MDV-3	HVT gB	SB-1-1F	5′	TGCCCCATACCGTTGAACAATTCCGCC	27	59051–59077 ^c^	483
		HVT-8R	5′	CGCCTCGTCCGGCAAGGCCATCTTGA	26	53806–53831 ^d^	

^a^ Reference strain GX0101 genome, GenBank Acc. No. JX844666. ^b^ CVI988 genome, GenBank Acc. No. DQ530348. ^c^ SB-1 genome, GenBank Acc. No. HQ840738. ^d^ HVT strain FC-126 genome, GenBank Acc. No. AF291866.

**Table 2:** Positive rates of MDV infection in 30 clinical cases, detected by the mPCR method.

No.	Poultry Farm	Breeds	Category	Positive Rates of Virus Infection											
				Spleen				Liver				Total			
				S-meq	L-meq	HVT	SB-1	S-meq	L-meq	HVT	SB-1	S-meq	L-meq	HVT	SB-1
1	HNZMD	Liangfenghua	Broiler	11/11 (100%)	0/11 (0%)	0/11 (0%)	0/11 (0%)	11/11 (100%)	0/11 (0%)	0/11 (0%)	0/11 (0%)	22/22 (100%)	0/22 (0%)	0/22 (0%)	0/22 (0%)
2	HNXZ1	Partridge chicken	Layer	5/6 (83.3%)	0/6 (0%)	0/6 (0%)	6/6 (100%)	5/6 (83.3%)	0/6 (0%)	0/6 (0%)	5/6 (83%)	10/12 (83.3%)	0/12 (0%)	0/12 (0%)	11/12 (91.6%)
3	HNZM	Partridge chicken	Broiler	1/12 (8.3%)	0/12 (0%)	0/12 (0%)	0/12 (0%)	0/12 (0%)	0/12 (0%)	0/12 (0%)	1/12 (8.3%)	1/24 (4.2%)	0/24 (0%)	0/24 (0%)	1/24 (4.2%)
4	HNXZ2	Liangfenghua	Layer	0/3 (0%)	0/3 (0%)	0/3 (0%)	0/3 (0%)	0/3 (0%)	0/3 (0%)	0/3 (0%)	0/3 (0%)	0/6 (0%)	0/6 (0%)	0/6 (0%)	0/6 (0%)
5	HNYY1	Jinghong	Layer	15/15 (100%)	0/15 (0%)	0/15 (0%)	14/15 (93.3%)	13/15 (86.6%)	0/15 (0%)	0/15 (0%)	12/15 (80%)	28/30 (93.3%)	0/30 (0%)	0/30 (0%)	26/30 (86.6%)
6	HNLK1	Hyline Brown	Layer	4/15 (26.6%)	1/15 (6.6%)	0/15 (0%)	13/15 (86.6%)	2/15 (13.3%)	0/15 (0%)	0/15 (0%)	11/15 (73.3%)	6/30 (20%)	1/30 (3.3%)	0/30 (0%)	24/30 (80%)
7	HNLK2	Jinghong	Layer	3/9 (33.3%)	1/9 (11.1%)	0/9 (0%)	7/9 (77.8%)	0/9 (0%)	0/9 (0%)	1/9 (11.1%)	8/9 (88.9%)	3/18 (16.6%)	1/18 (5.5%)	1/18 (5.5%)	15/18 (83.3%)
8	HNZC1	Jinghong	Layer	6/6 (100%)	0/6 (0%)	0/6 (0%)	5/6 (83.3%)	5/6 (83.3%)	0/6 (0%)	0/6 (0%)	4/6 (66.7%)	11/12 (91.6%)	0/12 (0%)	0/12 (0%)	9/12 (75%)
9	HNSQ1	Jinghong	Layer	11/12 (91.6%)	0/12 (0%)	0/12 (0%)	4/12 (33.3%)	10/12 (83.3%)	0/12 (0%)	0/12 (0%)	3/12 (25%)	21/24 (87.5%)	0/24 (0%)	0/24 (0%)	7/24 (29.1%)
10	HNLY1	Jinghong	Layer	4/4 (100%)	0/4 (0%)	0/4 (0%)	1/4 (25%)	4/4 (100%)	0/4 (0%)	0/4 (0%)	1/4 (25%)	8/8 (100%)	0/8 (0%)	0/8 (0%)	2/8 (25%)
11	HNYC1	Jinghong	Layer	8/9 (88.9%)	0/9 (0%)	0/9 (0%)	8/9 (88.9%)	4/9 (44.4%)	0/9 (0%)	0/9 (0%)	5/9 (55.5%)	12/18 (66.7%)	0/18 (0%)	0/18 (0%)	13/18 (72.2%)
12	SDCX1	Jinghong	Layer	9/9 (100%)	0/9 (0%)	0/9 (0%)	8/9 (88.9%)	9/9 (100%)	0/9 (0%)	0/9 (0%)	8/9 (88.9%)	18/18 (100%)	0/18 (0%)	0/18 (0%)	16/18 (88.9%)
13	SDSX	Jinghong	Layer	2/7 (28.5%)	0/7 (0%)	0/7 (0%)	5/7 (71.4%)	2/7 (28.5%)	0/7 (0%)	0/7 (0%)	2/7 (28.5%)	4/14 (28.5%)	0/14 (0%)	0/14 (0%)	7/14 (50%)
14	SDCW	Hyline Brown	Layer	9/11 (81.8%)	0/11 (0%)	0/11 (0%)	3/11 (27.3%)	9/11 (81.8%)	0/11 (0%)	0/11 (0%)	3/11 (27%)	18/22 (81.8%)	0/22 (0%)	0/22 (0%)	6/22 (27.2%)
15	SDCX2	Jinghong	Layer	6/7 (85.7%)	0/7 (0%)	0/7 (0%)	7/7 (100%)	5/7 (71.4%)	0/7 (0%)	0/7 (0%)	7/7 (100%)	11/14 (78.5%)	0/14 (0%)	0/14 (0%)	14/14 (100%)
16	HNSQ2	Jinghong	Layer	4/5 (80%)	0/5 (0%)	0/5 (0%)	3/5 (60%)	4/5 (80%)	0/5 (0%)	0/5 (0%)	3/5 (60%)	8/10 (80%)	0/10 (0%)	0/10 (0%)	6/10 (60%)
17	HNSC	Hyline Brown	Layer	13/14 (92.8%)	0/14 (0%)	0/14 (0%)	10/14 (71.4%)	13/14 (92.8%)	0/14 (0%)	0/14 (0%)	9/14 (64.2%)	26/28 (92.8%)	0/28 (0%)	0/28 (0%)	19/28 (67.8%)
18	HNYC2	Jinghong	Layer	13/14 (92.8%)	0/14 (0%)	0/14 (0%)	6/14 (42.8%)	9/14 (64.2%)	0/14 (0%)	0/14 (0%)	2/14 (14.2%)	22/28 (78.5%)	0/28 (0%)	0/28 (0%)	8/28 (28.5%)
19	HNZC2	Jinghong	Layer	11/12 (91.6%)	0/12 (0%)	0/12 (0%)	7/12 (58.3%)	11/12 (91.6%)	0/12 (0%)	0/12 (0%)	7/12 (58.3%)	22/24 (91.6%)	0/24 (0%)	0/24 (0%)	14/24 (58.4%)
20	HNXZ3	Partridge chicken	Breeder	5/8 (62.5%)	0/8 (0%)	0/8 (0%)	5/8 (62.5%)	4/8 (50%)	0/8 (0%)	0/8 (0%)	3/8 (37.5%)	9/16 (56.2%)	0/16 (0%)	0/16 (0%)	8/16 (50%)
21	HNQX	Jinghong	Layer	9/9 (100%)	0/9 (0%)	0/9 (0%)	3/9 (33.3%)	9/9 (100%)	0/9 (0%)	0/9 (0%)	2/9 (22.2%)	18/18 (100%)	0/18 (0%)	0/18 (0%)	5/18 (27.8%)
22	HNZC3	Jinghong	Layer	3/5 (60%)	0/5 (0%)	0/5 (0%)	4/5 (80%)	2/5 (40%)	0/5 (0%)	0/5 (0%)	4/5 (80%)	5/10 (50%)	0/10 (0%)	0/10 (0%)	8/10 (80%)
23	HNYY2	Jinghong	Layer	0/5 (0%)	3/5 (60%)	0/5 (0%)	1/5 (20%)	0/5 (0%)	1/5 (20%)	0/5 (0%)	0/5 (0%)	0/10 (0%)	4/10 (40%)	0/10 (0%)	1/10 (10%)
24	HNPDS	Hyline Brown	Layer	0/6 (0%)	0/6 (0%)	0/6 (0%)	6/6 (100%)	0/6 (0%)	0/6 (0%)	0/6 (0%)	6/6 (100%)	0/12 (0%)	0/12 (0%)	0/12 (0%)	12/12 (100%)
25	HNLY2	Jinghong	Layer	0/6 (0%)	0/6 (0%)	0/6 (0%)	4/6 (66.7%)	0/6 (0%)	0/6 (0%)	0/6 (0%)	2/6 (33.3%)	0/12 (0%)	0/12 (0%)	0/12 (0%)	6/12 (50%)
26	HNZC4	Hyline Brown	Layer	5/8 (62.5%)	0/8 (0%)	0/8 (0%)	6/8 (75%)	6/8 (75%)	0/8 (0%)	0/8 (0%)	6/8 (75%)	11/16 (68.7%)	0/16 (0%)	0/16 (0%)	13/16 (81.25%)
27	HNSX	Jinghong	Layer	5/5 (100%)	0/5 (0%)	0/5 (0%)	0/5 (0%)	5/5 (100%)	0/5 (0%)	0/5 (0%)	0/5 (0%)	10/10 (100%)	0/10 (0%)	0/10 (0%)	0/10 (0%)
28	HNZC5	Jinghong	Layer	5/7 (71.4%)	0/7 (0%)	0/7 (0%)	0/7 (0%)	5/7 (71.4%)	0/7 (0%)	0/7 (0%)	1/7 (14.2%)	10/14 (71.4%)	0/14 (0%)	0/14 (0%)	1/14 (7.1%)
29	HNFQ	Hyline Brown	Layer	1/11 (9%)	0/11 (0%)	0/11 (0%)	11/11 (100%)	0/11 (0%)	0/11 (0%)	0/11 (0%)	11/11 (100%)	1/22 (4.5%)	0/22 (0%)	0/22 (0%)	22/22 (100%)
30	HNWS	Muyuan Red	Layer	1/20 (5%)	7/20 (35%)	0/20 (0%)	18/20 (90%)	UA	UA	UA	UA	1/20 (5%)	7/20 (35%)	0/20 (0%)	18/20 (90%)
	Total	NA	NA	169/271 (62.4%)	12/271 (4.4%)	0/271 (0%)	165/271 (60.9%)	147/251 (58.6%)	1/251 (0.4%)	1/251 (0.4%)	127/251 (50.6%)	316/522 (60.5%)	13/522 (2.5%)	1/522 (0.2%)	292/522 (55.9%)

UA, liver sample unavailable. NA, not applicable.

**Table 3:** Comparison of the positive rates of MDV infection in clinical splenic samples detected by mPCR and PCR.

No.	Poultry Farm	Breeds	Category			Positive Rates			
				mPCR			PCR		
				meq Genes			meq Genes		
				S-meq	L-meq	S-meq + L-meq	S-meq	L-meq	S-meq + L-meq
1	HNSQ1	Jinghong	Layer	9/10 (90%)	0/10 (0%)	9/10 (90%)	9/10 (90%)	0/10 (0%)	9/10 (90%)
2	SDCW	Hyline Brown	Layer	7/10 (70%)	0/10 (0%)	7/10 (70%)	8/10 (80%)	0/10 (0%)	8/10 (80%)
3	HNSC	Hyline Brown	Layer	9/10 (90%)	0/10 (0%)	9/10 (90%)	9/10 (90%)	0/10 (0%)	9/10 (90%)
4	HNZC2	Jinghong	Layer	9/10 (90%)	0/10 (0%)	9/10 (90%)	9/10 (90%)	0/10 (0%)	9/10 (90%)
5	SDCX1	Jinghong	Layer	9/10 (90%)	0/10 (0%)	9/10 (90%)	9/10 (90%)	0/10 (0%)	9/10 (90%)
6	HNQX	Jinghong	Layer	10/10 (100%)	0/10 (0%)	10/10 (100%)	10/10 (100%)	0/10 (0%)	10/10 (100%)
7	HNYC2	Jinghong	Layer	10/10 (100%)	0/10 (0%)	10/10 (100%)	10/10 (100%)	0/10 (0%)	10/10 (100%)
8	HNWS	Muyuan Red	Layer	1/10 (10%)	5/10 (50%)	6/10 (60%)	1/10 (60%)	5/10 (0%)	6/10 (60%)
9	HNZMD	Liangfenghua	Broiler	10/10 (100%)	0/10 (0%)	10/10 (100%)	10/10 (100%)	0/10 (0%)	10/10 (100%)
10	HNFQ	Hyline Brown	Layer	1/10 (10%)	0/10 (0%)	1/10 (10%)	1/10 (10%)	0/10 (0%)	1/10 (10%)
	Total			75/100 (75%)	5/100 (5%)	80/100 (80%)	76/100 (76%)	5/100 (5%)	81/100 (81%)

## Data Availability

The data supporting the conclusions of this article are included within the article. The raw data are available upon request.

## Supplementary Materials

The following supporting information can be downloaded at: https://www.mdpi.com/article/10.3390/v18040471/s1, Table S1: Background of the meq and *gB* genes of 18 reference MDV strains used for primer designment; Table S2: Primers designed for the amplifications of MDV-1 meq, MDV-2 *gB* and HVT *gB* genes; Figure S1: PCR amplification of MDV-1 meq genes for testing the specificities of different primer combinations; Figure S2: PCR identification of positive control plasmids using meq or *gB* specific primers.; Figure S3: Optimization and specificity of different primer combinations determined by mPCR amplification; Figure S4: Schematic diagram of primer pairs meq-2F/2R specific to MDV-1 meq genes; Figure S5: Schematic diagram of primer pairs SB-1-1F/1R specific to SB-1 *gB* genes; Figure S6: Schematic diagram of primer pairs SB-1-1F/HVT-8R specific to HVT *gB* genes; Figure S7: MDV infection in clinical samples collected from representative poultry farms detected by mPCR.
