# Supplementary material for: Development of a Multiplex PCR Method for Efficient Differential Diagnosis of Clinical Cases and Vaccine Immunization of Marek’s Disease
Source: Viruses. 2026 Apr 16;18(4):471. doi: 10.3390/v18040471 (PMC13120613; doi:10.3390/v18040471)
Supplement: Supplementary file 1 [file viruses-18-00471-s001.zip › viruses-4239962-supplementary.pdf]

Supplementary tables

Table S1. Background of the *meq* and *gB* genes of 18 reference MDV strains used for primer designment.

Serotype	No.	Pathotype *	Strain	GenBank Acc. No.	
				meq	gB
MDV-1	1	vv+	648A-P101	AY362725.1	NA
	2	vv	J-1	HQ190957.1	NA
	3	vv	GX0101	JX844666.1	NA
	4	vv	Md5	AF243438.1	NA
	5	vv	CC/1409	KU744560.1	NA
	6	vv	LCC	KP888815.1	NA
	7	vv	LTS	KP888838.1	NA
	8	vv	WC/1203	KP888839.1	NA
	9	vv	JL/1404	KU744559.1	NA
	10	vv	LCY	HQ658627.1	NA
	11	vv	HNLC503	HF546096.1	NA
	12	vv	RB-1B	EF523390.1	NA
	13	v	GA	AF147806.2	NA
	14	m	CVI988	AY164640.1	NA
	15	m	814	JF742597.1	NA
	16	m	CU-2	AY362708.1	NA
MDV-2/SB-1	17	NA	SB-1	NA	HQ840738
MDV-3/HVT	18	NA	FC-126	NA	AF291866.1

* m, mild MDV; v, virulent MDV; vv, very virulent MDV; vv+; very virulent plus MDV; NA, not applicable.

Table S2. Primers designed for the amplifications of MDV-1 *meq*, MDV-2 *gB* and HVT *gB* genes.

Serotype	Target*	No.	Primer name	Type	Sequence (5′-3′)	Length (nt)	Genomic location**
**MDV-1**	L-meq or S-meq	1	meq-2F	5′	ATGTCTCAGGAGCCAGAGCCGGGCGCTAT	29	133603-133631
		2	meq-2R	5′	TCAGGGTCTCCCGTCACCTGGAAACCACCA	30	134593-134622
		3	meq-3F	5′	TCACCGGATGAACCTAACGCTCCACATTGC	30	134068-134097
		4	meq-3R	5′	TGGGGAGGGCAGAAGAGGGAATGG	24	134342-134319
		5	meq-4R	5′	AGATGGAGGCTGGGGAGGGCAGAAGAGGGAATGG	34	134352-134319
		6	meq-5F	5′	GAATCGTGACGCCTCTC	17	133800-133816
		7	meq-5R	5′	GCTGAGCGTAAACCGTCCCCGGCGAT	26	134431-134406-
		8	meq-6F	5′	CATGTGAAGAGCTGCA	16	133865-133880
		9	meq-6R	5′	AACCACCAGACCGTAGACT	19	134600-134582
		10	meq-7F	5′	CCCTACAGTCCCGCTGACGATCCGT	25	133633-133657
		11	meq-7R	5′	CCTTTAACCCTTTCCTTTATGTTGATCTTCCCG	33	134740-134708
		12	meq-8F	5′	CTTCAATACTTTCGGGTCTGTGGGTGT	27	133438-133464
		13	meq-8R	5′	ATGTCAGTAAATCGATAAATAATGC	25	134764-134740
		14	meq-9F	5′	TGCTGGAATGTTAAGAATAAATTCCGCAC	29	133502-133530
		15	meq-9R	5′	CCCTGACCATGTAACTCAAAATAGTTCTTCCG	32	134845-134814
		16	meq-10R	5′	GAACTCCTGGAGCCAACAAATCCCCTG	27	134867-134841
		17	meq-11R	5′	TTATCTCATACTTCGGAACTCCTGG	25	134882-134858
		18	meq-12R	5′	TTCCACATAGCTAAGTTTATCTCAT	25	134898-134874
**MDV-2**	SB-1 gB	1	SB-1-1F	5′	TGCCCCATACCGTTGAACAATTCCGCC	27	59051-59077
		2	SB-1-1R	5′	GCAGAATTCCATGAGGGTCGC	21	59533-59513
		3	SB-1-2F	5′	GCAACCTCTACCATCTTAACAAAGTTATAGT	31	59297-59327
		4	SB-1-2R	5′	GCATGCTCTTTCCAATAAAACAAATG	26	59899-59874
		5	SB-1-3F	5′	TCCCTAAGGTAGCGCTCGCG	20	59613-59632
		6	SB-1-3R	5′	TAGAACAATTTAGGCAGATAGACG	24	60404-60381
		7	SB-1-4F	5′	CGTCTATCTGCCTAAATTGTTCTA	24	60381-60404
		8	SB-1-4R	5′	GGTTTTCTACTTCTTCGGCCGAGTCG	26	61111-61086
**MDV-3**	HVT gB	1	HVT-1F	5′	CCGTCTCAGAATCCGTGTCGTT	22	52567-52588
		2	HVT-1R	5′	CGATGGATCTGACAGTGAATTGATG	25	52769-52793
		3	HVT-2R	5′	CCGCGTACCGATGGATCTGACAGT	24	52801-52778

4	HVT-3F	5′	TGAATCGATCTCAATACATGAT	22	53540-53561
5	HVT-3R	5′	CCATCGCGACCTCAGAAATTAAATT	25	55095-55071
6	HVT-4F	5′	AGAATCTGAATCGATCTCAATACATGAT	28	53534-53561
7	HVT-4R	5′	CCAATCTTATCCATCGCGACCTCAGAAATTAAATT	35	55105-55071
8	HVT-5F	5′	TTGCCGGACGAGGCGCGAT	19	53817-53835
9	HVT-5R	5′	CTGCGTGCTAGAGTGGGCAAG	21	54217-54197
10	HVT-6F	5′	TCAAGATGGCCTTGCCGGACGAGGCG	26	53806-53831
11	HVT-6R	5′	ACGGAAATGCTGCGTGCTAGAGTGGGCAAG	30	54226-54197
12	HVT-7R	5′	ATCGCGCCTCGTCCGGCAAG	20	53835-53816
13	HVT-8R	5′	CGCCTCGTCCGGCAAGGCCATCTTGA	26	53806-53831

* *L-Meq*, long *meq* gene encoded by virulent MDV-1 strains; *S-Meq*, short *meq* gene encoded by vaccine MDV-1 strains. ** Reference MDV-1 strain GX0101 genome, GenBank Acc. No. JX844666; MDV-2 strain SB-1 genome, GenBank Acc. No. HQ840738; MDV-3/HVT strain FC-126 genome, GenBank Acc. No. AF291866.

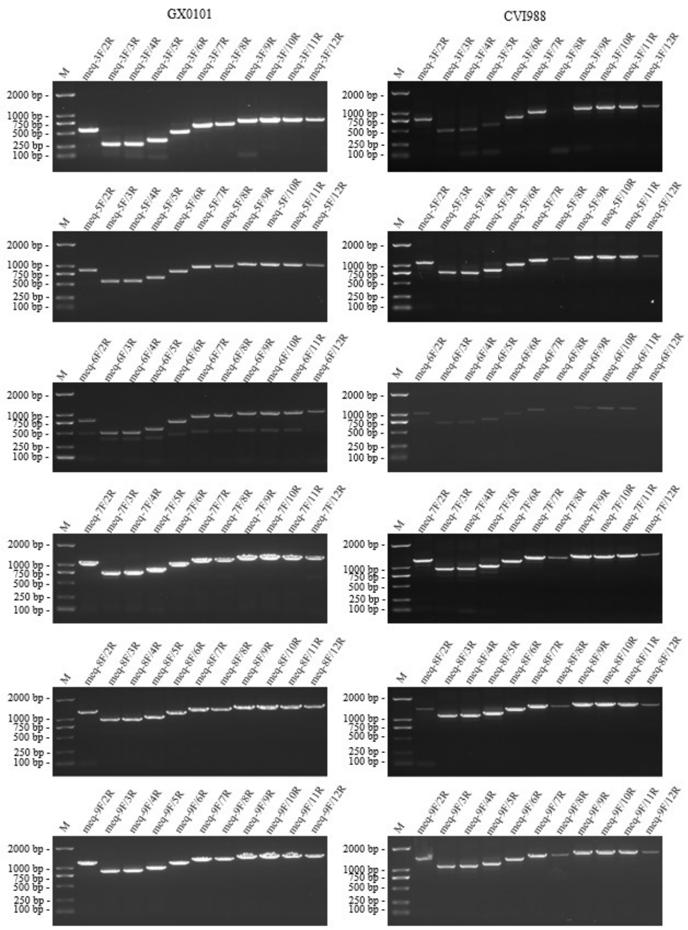

Figure S1. PCR amplification of MDV-1 *meq* genes for testing the specificities of different primer combinations. The virulent GX0101 (left) and vaccine CVI988 (right) viruses were used as DNA templates, respectively. The primer pair are abbreviated, such as meq-3F/2R shortened from meq-3F and meq-2R. M, DNA marker.

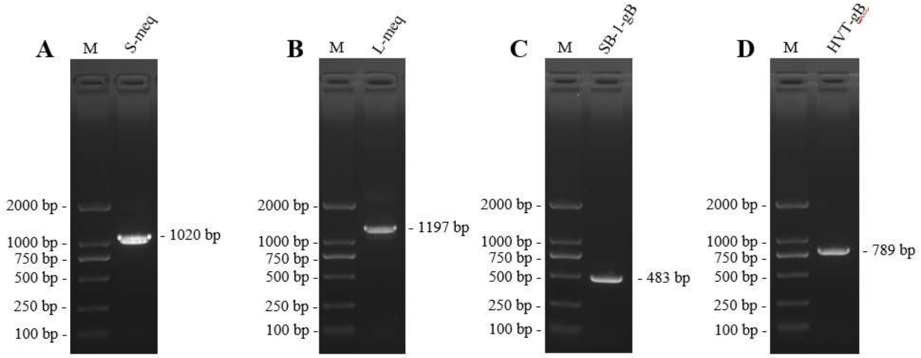

Figure S2. PCR identification of positive control plasmids using meq or gB specific primers. (A) plasmid pMD19-T-S-meq; (B) plasmid pMD19-T-L-meq; (C) plasmid pMD19-T-SB-1gB; (D) plasmid pMD19-T-HVTgB. M, DNA marker.

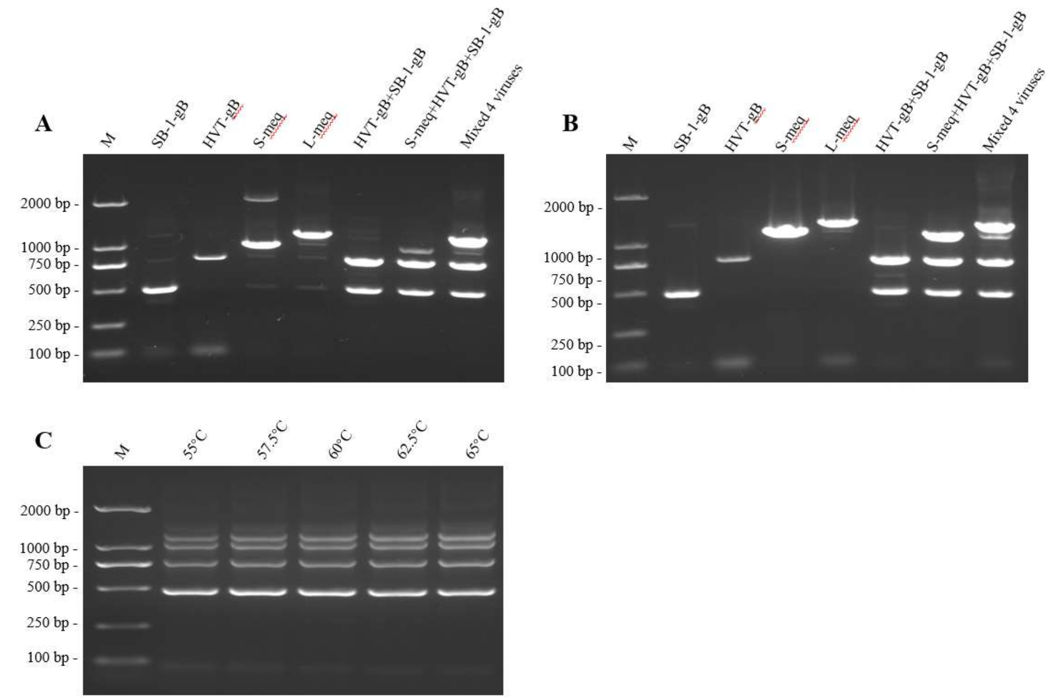

Figure S3. Optimization and specificity of different primer combinations determined by mPCR amplification. (A) Primer combination of meq-5F/7R+SB-1-1F/1R+HVT-8R and positive control plasmids used as DNA templates; (B) Primer combination of meq-7F/7R+SB-1-1F/1R+HVT-8R and positive control plasmids used as DNA templates; (C) Optimization of the annealing temperature for primer combination meq-2F/2R+SB-1-1F/1R+HVTgB-8R. M, DNA marker.

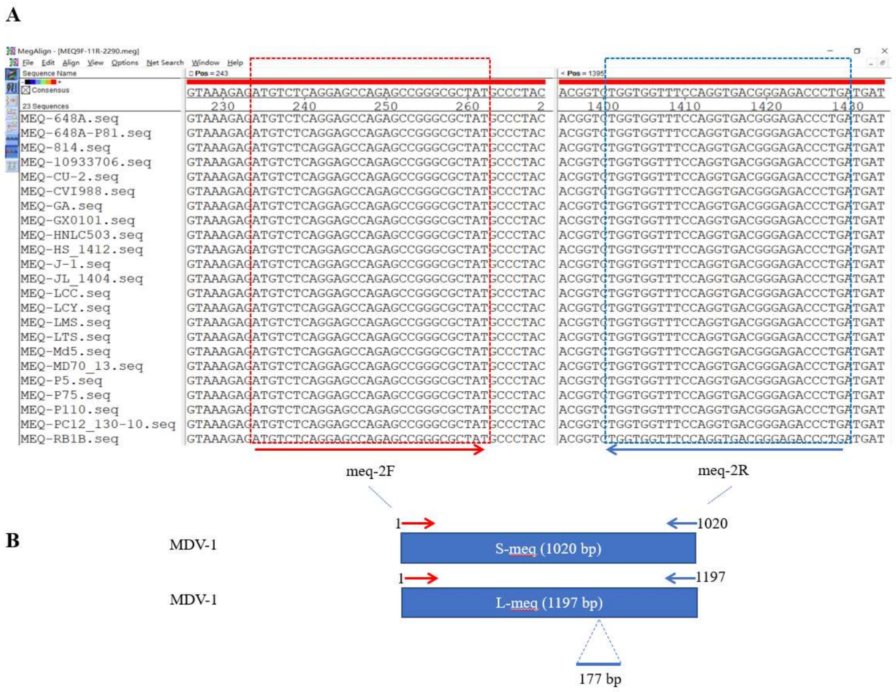

Figure S4. Schematic diagram of primer pairs meq-2F/2R specific to MDV-1 *meq* genes. (A) Alignment of *meq* genes from different serotypes of MDV with differ virulence; (B) Schematic of amplicons of the long (L) and short (S) *meq* genes. Upper and bottom target sequences are shown by red or blue dashed boxes and arrows, respectively.

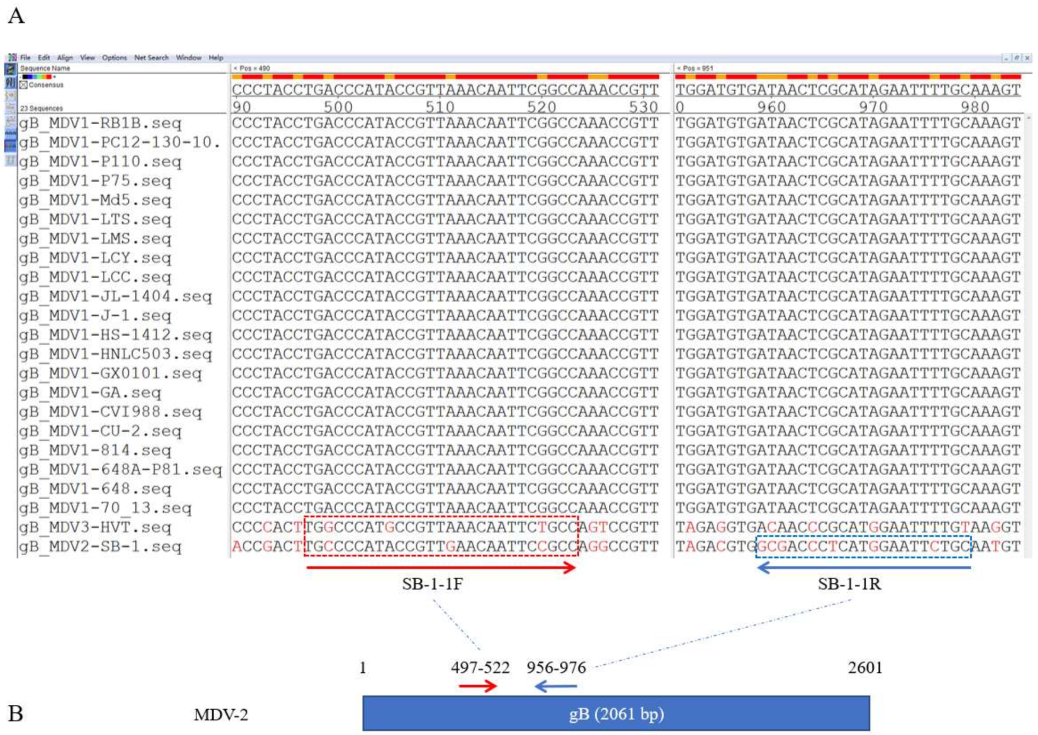

Figure S5. Schematic diagram of primer pairs SB-1-1F/1R specific to SB-1 *gB* genes. (A) Alignment of *gB* genes from different serotypes of MDV with differ virulence; (B) Schematic of amplicons of SB-1 *gB* gene. Upper and bottom target sequences are shown by red or blue dashed boxes and arrows, respectively.

A

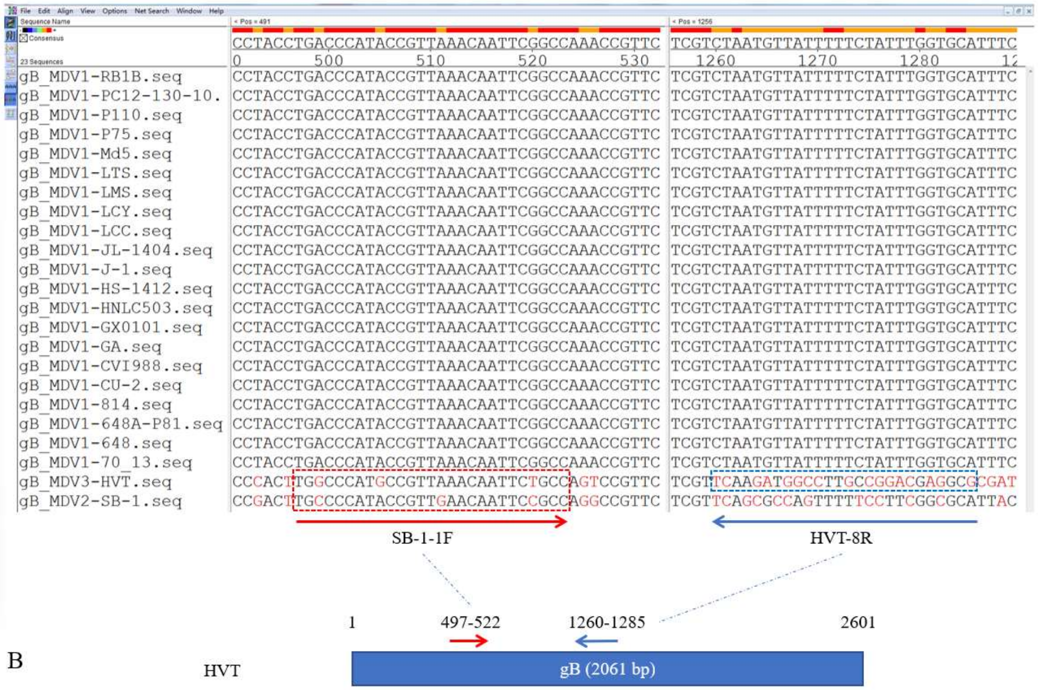

B

Figure S6. Schematic diagram of primer pairs SB-1-1F/HVT-8R specific to HVT *gB* genes. (A) Alignment of *gB* genes from different serotypes of MDV with differ virulence; (B) Schematic of amplicons of HVT *gB* gene. Upper and bottom target sequences are shown by red or blue dashed boxes and arrows, respectively.

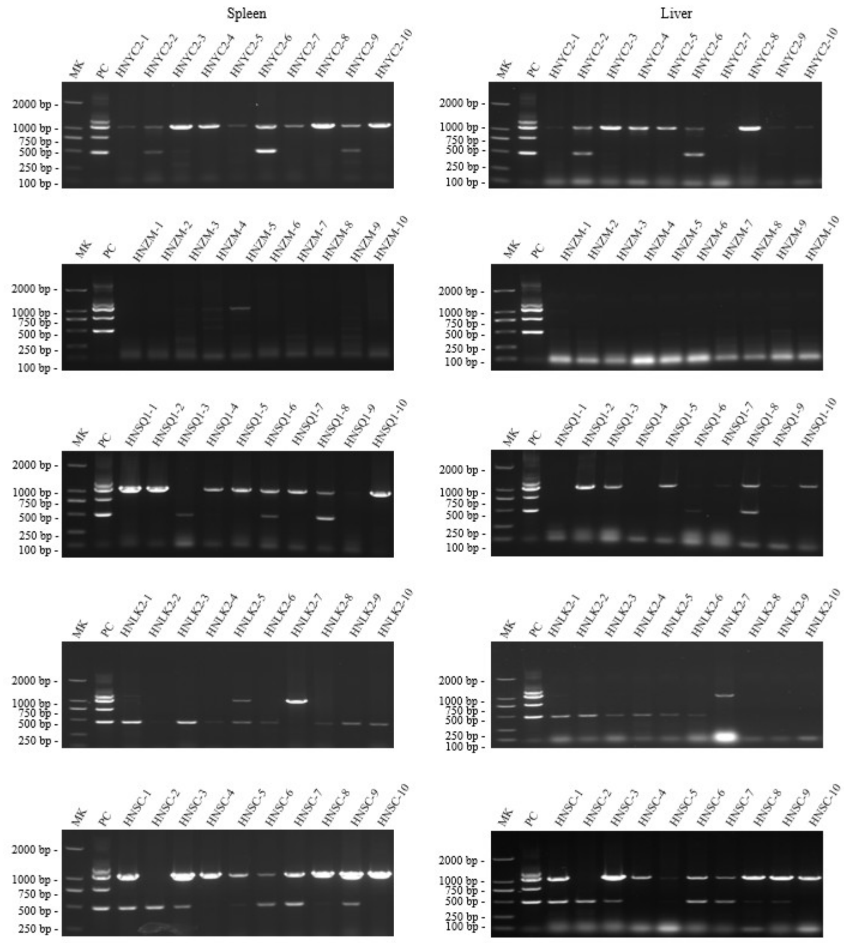

Figure S7. MDV infection in clinical samples collected from representative poultry farms detected by mPCR. M, DNA marker; PC, positive control plasmids. HNYC2, the 2nd case in Yucheng, Henan; HNZM, Zhongmu, Henan; HNSQ, Shangqiu, Henan; HNLK, Lankao, Henan; HNSC, Shangcai, Henan.
